# Catamenial Rectus Abdominis Pain Associated with Scar Endometriosis Status-post Low Transverse Cesarean Section

**DOI:** 10.7759/cureus.3778

**Published:** 2018-12-26

**Authors:** Paul Wasserman, Chandana Kurra, Kristin Taylor, Brian Wells, Ashlesha Sharma, Amie Leon

**Affiliations:** 1 Radiology, University of Florida, Jacksonville, USA

**Keywords:** scar endometriosis, catamenial, scar, endometriosis, rectus abdominis muscle

## Abstract

Endometriosis is the presence of endometrial tissue outside of the uterine cavity. Scar endometriosis (SE), a rare occurrence, results from inadvertent extrapelvic transplantation of endometrial tissue to an incision site, such as from a low transverse cesarean section (LTCS). The reported incidence of abdominal wall scar endometriosis status-post cesarean section is 0.03 - 0.6%. We present a case of rectus abdominis scar endometriosis diagnosed four years following an LTCS. Our case report discusses the history/presentation, imaging findings, histopathology, and pertinent literature concerning abdominal wall scar endometriosis.

## Introduction

While endometriosis is a common disease, ectopic endometrial implants involving the abdominal wall, pleura, surgical scars, and extremities are much less common [1]. Given the rare occurrence of abdominal wall scar endometriosis (0.03 - 0.6%), we report a case of intramuscular rectus abdominis incisional endometriosis in a 34-year-old female, diagnosed four years following a low transverse cesarean section (LTCS) [1]. Patients presenting with lower abdominal wall pain with cyclical swelling of a palpable mass post-gynecological surgery should raise the clinician’s index of suspicion for scar endometriosis (SE). A common presentation of SE is pain and swelling at the surgical scar site. The clinical presentation has been known to vary from as short as a few months to over 10 years. In our case, the patient presented four years after the LTCS. The diagnosis of SE, in our case, depended on a high degree of suspicion based on clinical history and examination, with imaging and histopathology playing a role in confirming the final diagnosis.

## Case presentation

We report the case of a 34-year-old, multigravid female who presented to the Obstetrics/Gynecology Clinic with complaints of right-sided pelvic pain and a palpable “knot” within the right anterior pelvic wall region. She reported first noticing this mass four years prior, following her cesarean section, and attributed this to scar tissue from her LTCS incision. The patient reported a two-month history of focal increased tenderness at the site, particularly with menstruation, and described the discomfort as constant, dull, and aching. Her pain started around the mass and radiated towards her back.

Physical examination revealed normal vital signs and an abdominal right lower quadrant hard, non-mobile, tender-to-touch, 1.6 cm mass palpated 3 - 4 cm cranial to her LTCS cutaneous scar.

**Figure 1:**
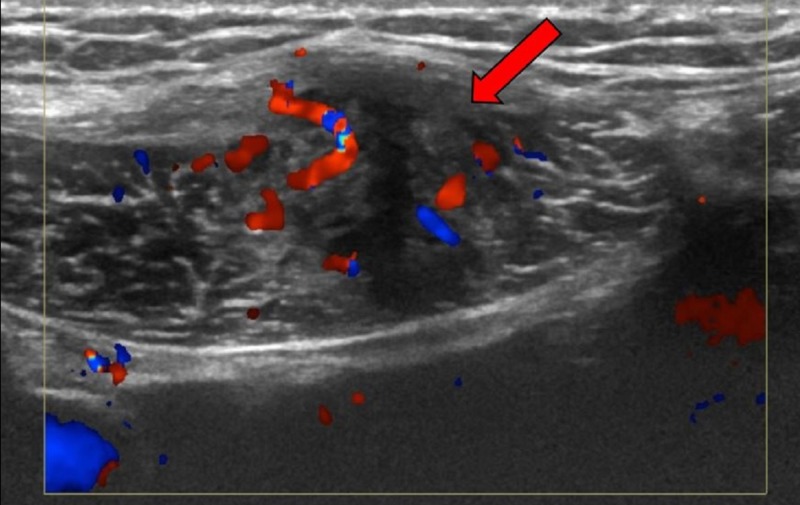
Pelvic ultrasound Ultrasound of the right lower quadrant anterior abdominal wall demonstrates a complex intramuscular mass with internal vascularity (as evidenced by the red and blue Doppler signal) in the lower right rectus abdominis muscle

The palpable mass was further evaluated with an ultrasound (US) which revealed a 1.6 cm complex, predominantly hypoechoic, intramuscular mass exhibiting foci of internal vascularity and punctate echogenic foci (Figure 1). The imaging differential diagnostic considerations included an inflammatory process, hematoma, or soft tissue mass.

**Figure 2:**
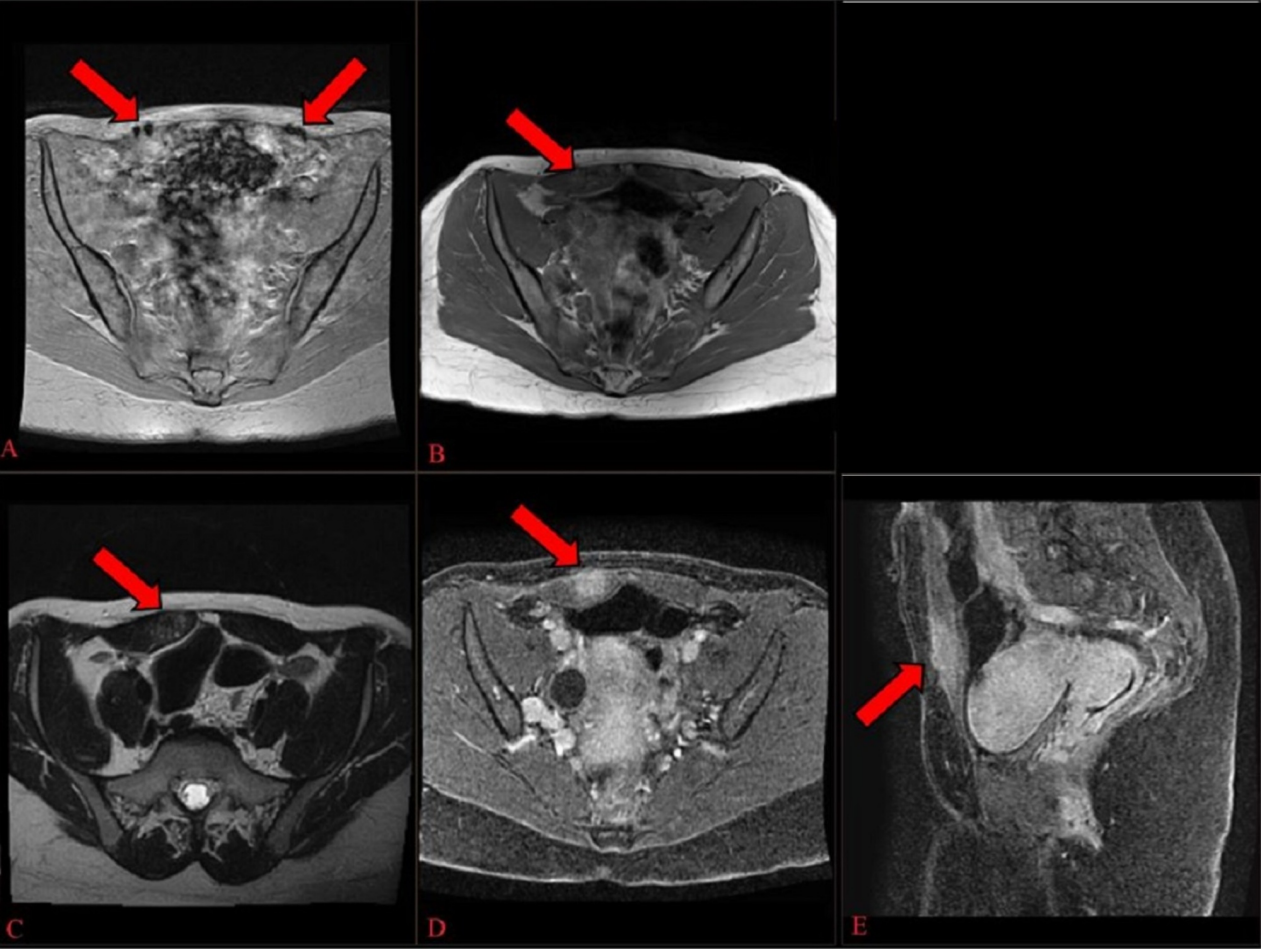
Magnetic resonance imaging of the pelvis A) Gradient image demonstrates "blooming" artifact overlying the rectus abdominis muscle corresponding to patient's low transverse cesarean section incision site; B, C) T1 and T2-weighted axial unenhanced images demonstrate a heterogenous mass in the right rectus abdominis musculature; D, E) Post-contrast T1-weighted gradient echo images demonstrate enhancement of the mass.

Subsequently, magnetic resonance imaging (MRI) of the pelvis with and without intravenous gadolinium contrast was performed to better characterize the abnormality. The MRI revealed a 4 cm area of mildly heterogenous T1 and T2 signals with enhancement after the administration of gadolinium contrast. The mass was located in the medial aspect of the right rectus abdominis muscle, immediately adjacent to a curvilinear area of magnetic susceptibility artifact, which corresponded to the LTCS incision (Figure 2). Differential diagnostic considerations included scar endometriosis (particularly, given our patient’s history), scar/granulation tissue, desmoid tumor, lymphoma, focal muscle strain, and, less likely, metastatic disease.

**Figure 3:**
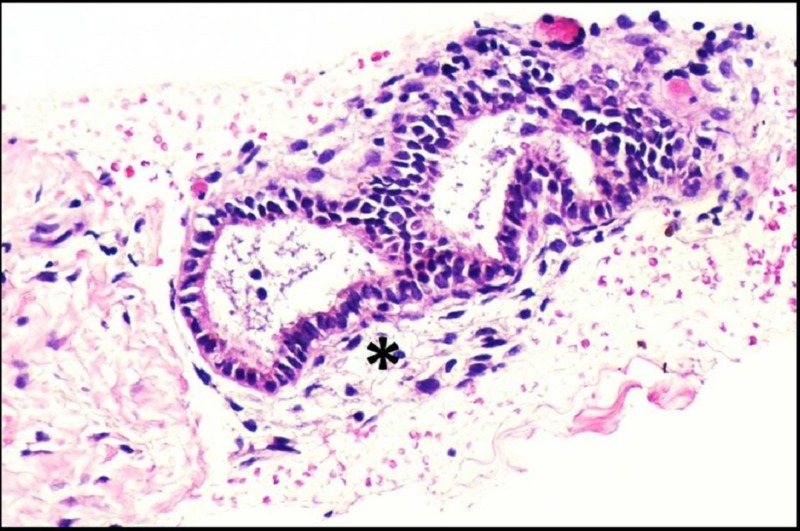
Pathology Core biopsy reveals the characteristic findings of endometrial glands surrounded by stroma (exemplified by the asterisk). Hematoxylin and eosin stain; magnification x200

As a result, a US-guided percutaneous core biopsy of the right rectus abdominis soft tissue mass was performed using an 18-gauge spring-loaded coaxial biopsy system. The histopathology demonstrated the presence of endometrioid glands surrounded by endometrial stroma (Figure 3). The glands were lined by columnar epithelial cells and the stroma was composed of small, bland fusiform cells with scant cytoplasm. For the diagnosis of endometriosis, two of the three characteristic findings (extrauterine foci of endometrial glands with variable amounts of stroma and hemorrhage) were present [2].

Surgery to resect the ectopic endometrial tissue and to perform lysis of the adhesions of the LTCS incision area was subsequently performed.

## Discussion

Scar endometriosis is described in the literature but remains an extremely rare diagnosis with its incidence in post-cesarean scar tissue estimated at 0.03% - 0.4% [3-4] and up to 0.6% [1]. The etiology of SE has been attributed to a transportation theory in the literature [1]. The transportation theory suggests that endometrial cells are inadvertently transported to extrauterine areas, such as incision sites, where, in some instances, endometriosis develops and is affected by the cyclical hormonal changes of menses [1, 5].

Diagnosing SE can be challenging. As described in published case reports, often there is a history of pain that corresponds to the patient’s menstrual cycles [6]. Other differential diagnoses, such as hematoma, abscess, hernia, scar tissue, or even malignancy, should be considered and pursued where clinically warranted [7].

The imaging appearance of scar endometriosis depends on the menstrual cycle phase and duration of the process [8]. Ultrasound is often nonspecific, and our case revealed a solid, hypoechoic, vascularized soft tissue nodule with spiculated margins infiltrating the surrounding muscle tissue (Figure 1) [5]. Computed tomography (CT) and MRI have utility, although CT without contrast is limited due to the lack of soft tissue contrast. The MRI findings of scar endometriosis can vary due to the chronicity of the blood products. Our case revealed an enhancing mass that exhibited a heterogeneously isointense to hyperintense T1 and T2-weighted appearance (Figure 2) [8]. The diagnosis of scar endometriosis, in this case, was suggested by a thorough review of the patient’s history (specifically, that her symptoms were catamenial in nature). Likewise, the close proximity of a magnetic susceptibility artifact, attributed to the incision site, also heightened the suspicion for scar endometriosis. Despite the imaging findings, a biopsy was necessary to confirm the diagnosis.

SE can be treated medically and surgically [9-10]. Medical treatments aim to create a hypoestrogenic environment to decrease hormonal stimulation of endometriosis. Low-dose estrogen oral contraceptives have been used to decrease pain from endometriosis and limit cell growth. Progestogens and danazol have been tried; however, they have not been proven to be effective [11-13].

Given the failure of medical therapy, surgical excision remains the treatment of choice and is curative for extrapelvic endometriosis in the vast majority of cases. For example, in a series of 21 patients with an average complaint duration lasting 25.3 months, recurrence was not observed in any patient during an average follow-up of 31.3 months after total excision [10]. The most frequent location of SE is right of the midline, along the cesarean-section Pfannenstiel incision, with the average size of the mass measuring 3 cm. Local wide excision with at least a 1 cm margin is considered the best clinical practice considering the possibilities of recurrence (4.3%) and malignant degeneration (0.3% - 1%) [14]. In addition, invasion of the abdominal wall musculature requires an en bloc resection of the underlying myofascial elements, raising the possibility of the formation of a hernia. Percutaneous cryoablation can also be used for effective local control [15]. The risk of abdominal wall hernia formation should be addressed and the possibility of a mesh repair should be explained to the patient. Wasfie et al. strongly recommended that, at the conclusion of a cesarean section, the abdominal wall wound should be cleaned thoroughly and irrigated vigorously with a high-jet saline solution before closure to avoid SE [16].

## Conclusions

While SE remains a rare disease, the diagnosis can be suggested when there is a focal mass in close proximity to a cesarean section scar exhibiting catamenial symptomatology. Appropriately tailored imaging can help to narrow the differential diagnoses in postoperative patients experiencing unusual postoperative abdominal wall pain.    
